# Treatment with AMD3100 attenuates the microglial response and improves outcome after experimental stroke

**DOI:** 10.1186/s12974-014-0232-1

**Published:** 2015-02-07

**Authors:** Helene L Walter, Gerlinde van der Maten, Ana Rita Antunes, Tadeusz Wieloch, Karsten Ruscher

**Affiliations:** Laboratory for Experimental Brain Research, Division of Neurosurgery, Department of Clinical Sciences, Lund University, BMC A13, S-22184 Lund, Sweden; Department of Neurology, University Hospital Cologne, Kerpener Straße 62, 50937 Cologne, Germany

**Keywords:** Chemokine receptor, CX3CR1, Cytokine, Fractalkine, Inflammation, Microglia, Stroke recovery

## Abstract

**Background:**

Recovery of lost neurological function after stroke is limited and dependent on multiple mechanisms including inflammatory processes. Selective pharmacological modulation of inflammation might be a promising approach to improve stroke outcome.

**Methods:**

We used 1,1′-[1,4-phenylenebis(methylene)]bis[1,4,8,11-tetraazacyclotetradecane] (AMD3100), an antagonist to the C-X-C chemokine receptor type 4 (CXCR4) and potential allosteric agonist to CXCR7, administered to mice twice daily from day 2 after induction of photothrombosis (PT). In addition to functional outcome, the dynamics of post-stroke microglia response were monitored *in vivo* by 2-photon-laser-microscopy in heterozygous transgenic CX_3_CR1-green fluorescent protein (GFP) mice (CX_3_CR1^GFP/+^) and complemented with analyses for fractalkine (FKN) and pro-inflammatory cytokines.

**Results:**

We found a significantly enhanced recovery and modified microglia activation without affecting infarct size in mice treated with AMD3100 after PT. AMD3100 treatment significantly reduced the number of microglia in the peri-infarct area accompanied by stabilization of soma size and ramified cell morphology. Within the ischemic infarct core of AMD3100 treated wild-type mice we obtained significantly reduced levels of the endogenous CX_3_CR1 ligand FKN and the pro-inflammatory cytokines interleukin (IL)-1β and IL-6. Interestingly, in CX_3_CR1-deficient mice (homozygous transgenic CX_3_CR1-GFP mice) subjected to PT, the levels of FKN were significantly lower compared to their wild-type littermates. Moreover, AMD3100 treatment did not induce any relevant changes of cytokine levels in CX_3_CR1 deficient mice.

**Conclusion:**

After AMD3100 treatment, attenuation of microglia activation contributes to enhanced recovery of lost neurological function in experimental stroke possibly due to a depression of FKN levels in the brain. We further hypothesize that this mechanism is dependent on a functional receptor CX_3_CR1.

## Background

After ischemic stroke, the brain may regain lost neurological function to a limited extent by compensation and relearning [1,2]. The definite outcome can be improved by established rehabilitation regimes and might be enhanced by supportive pharmacological treatments.

Parallel and partially opposite processes contribute to restoration of brain function after stroke. The interaction of multiple mechanisms including neuronal plasticity, astrogliosis, cell genesis, angiogenesis and inflammation are important for the clinical development. Interestingly, anti-inflammatory treatment significantly contributes to functional recovery; examples are the specific blockade of chemokines or pro-inflammatory molecules and their receptors. We have shown that modulation of the C-X-C chemokine receptor type 4/C-X-C chemokine receptor type 7 (CXCR4/CXCR7) pathway significantly improves functional outcome and is associated with a reduction of T-helper cells in the ischemic hemisphere [3]. Also, treatment with specific recombinant T cell receptor ligands [4] has been shown to improve recovery after stroke.

Selective modulation of microglia function could be a promising strategy that has been addressed in several experimental studies [5-7]. Recent investigations suggest that the C-X-C chemokine ligand 12 (CXCL12)/CXCR4/CXCR7 and the fractalkine (FKN)/CX_3_CR1 pathways are instrumental in the regulation of microglia [8]. CXCL12 stimulates the release of FKN, a chemokine that, in the brain, is mainly expressed by neurons [9,10] and exists in a membrane-bound and a soluble form [11-13]. While membranous FKN is involved in mechanisms of cell adhesion, the soluble form acts as a chemoattractant [10]. The sole receptor for FKN is CX_3_C chemokine receptor 1 (CX_3_CR1) and is expressed on different immune cell populations including microglia [9,14-16]. Initial studies have demonstrated that the FKN/CX_3_CR1 pathway is instrumental in chemotaxis of microglia [8,9], leukocyte trafficking and microglia/macrophage recruitment [12]. In *in vitro* assays, FKN showed neuroprotective properties by activation of neuronal cell survival pathways [17], and FKN also led to an increase of microglia survival [18].

After stroke, FKN expression increases in the necrotic infarct core within the first hours followed by an upregulation in the peri-infarct area within the first week after onset of the insult. CX_3_CR1-positive cells can be detected in the ischemic tissue from 24 hours and are strongly upregulated with a peak at day 7 after stroke [12]. CX_3_CR1-deficient mice subjected to transient middle cerebral artery occlusion (MCAO) showed smaller lesions compared to heterozygote and wild-type littermates accompanied by reduced levels of pro-inflammatory cytokines [19]. Results have been confirmed showing that the administration of FKN to wild-type mice resulted in smaller infarcts, but administration of FKN to FKN-deficient mice resulted in bigger infarcts in a model of permanent MCAO. Moreover, application of FKN to microglia cell cultures resulted in reduced phagocytosis [20]. Summarizing these findings, administration of FKN but also CX_3_CR1 deficiency seems to be beneficial in the acute phase after stroke. However, the role of this pathway during the first weeks after stroke has not been investigated.

Based on our previous studies demonstrating that antagonism of CXCR4 by 1,1′-[1,4-phenylenebis(methylene)]bis[1,4,8,11-tetraazacyclotetradecane] (AMD3100) enhanced functional recovery after experimental stroke [3] we hypothesized that specific CXCR4 antagonism could be a therapeutic target to affect the FKN/CX_3_CR1 pathway and to modulate microglia function after stroke, to finally improve long-term outcome after permanent focal ischemia.

## Methods

### Experimental design

**Figure 1:**
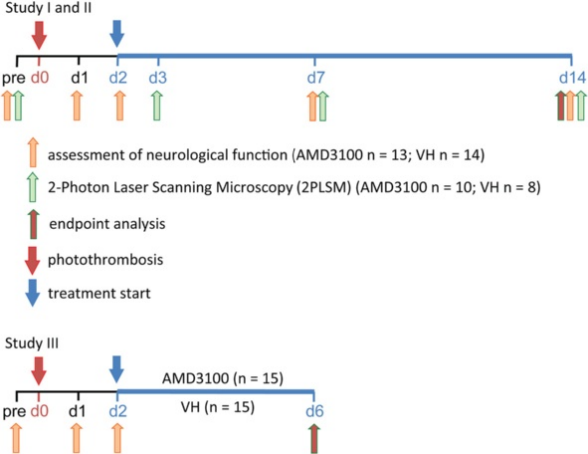
**Experimental design.** Assessment of neurological function was performed before (pre), and at day (d)1, 2, 7 and 14 following experimental stroke induced by photothrombosis (PT) (study I); 2-photon-laser-microscopy (2PLSM) was performed before and on days 3, 7 and 14 after PT (study II). One group of animals was treated with AMD3100 (0.5 mg/kg, twice daily), and control animals received intraperitoneal saline injections for the same time (vehicle (VH) twice daily). At the endpoint of the study (day 14), animals were sacrificed and brains processed for endpoint analyses. Fractalkine and cytokine levels were determined in CX_3_CR1 wild-type, heterozygote and knockout mice, respectively, on day 6 after PT (study III).

The microglial response and recovery of neurological function were observed in parallel until 14 days after ischemic stroke (Figure 1). Neurological function was assessed in wild-type male C57BL/6 N-mice (28 to 35 g, aged 12 to 16 weeks, purchased from Charles River, Sulzfeld, Germany) using a newly assembled neuroscore with a maximum score of 22 points (Table 1) consisting of paw placement, whisker-reflex and tail suspension, as well as a combination of the foot-fault test [21] and the ladder-rung test [22]. The animals were trained at least three times in climbing the ladder before recording the neurological status prior to stroke. Animals were excluded from the study if the full score was not achieved before photothrombosis (PT) (neuroscore <22 points) or if only insufficient neurological deficits were observed at day 1 after PT (neuroscore >11 points). Permanent cerebral ischemia was induced by PT. Mice were treated with either AMD3100 or saline vehicle (vh) from day 2 to day 14, administered intraperitoneally twice daily (every 12 hours, 0.5 mg/kg body weight). Neurological deficits were assessed on days 1, 2, 7 and 14. Mice were randomized (every other animal was allocated to the same treatment group) and the studies were performed in a blinded fashion to the investigator who performed surgeries and behavioral tests. Microglial response was monitored *in vivo* by 2-photon-laser-microscopy (2PLSM) through a thinned skull-window before stroke, and at days 3, 7 and 14 (Figure 1). On day 14, animals were sacrificed and endpoint analysis was carried out by immunohistochemistry.

**Table 1:** **Composite neuroscore for quantification of neurological deficits before and after photothrombotic stroke at days 1, 2, 7 and 14**

**Test**	**Score and description**
Left forelimb flexion in suspension	0 - impaired paw is not moving and tightly held to the trunk
	1 - impaired paw mostly held to the trunk, slightly movable
	2 - impaired paw moves up and down, asymmetry to other paw
	3 - slight asymmetry between the paws, both paws well movable
	4 - normal, symmetric
Paw placement of the left side	0 - paw totally immobilized, hanging down, no movement
	1 - paw hanging, but little movement back and forth (horizontal plane)
	2 - paw hanging, horizontal and vertical movement below table level
	3 - paw hanging horizontal and vertical movement up to table surface level
	4 - paw hanging, mobile in all directions, reaching the table surface
	5 - normal, paw is immediately taken up to the table surface
Whisker-reflex, left side	0 - not present
	2 - present
Faults of the left forelimb walking 27 steps on a grid, 46.5 cm long, step size 2 mm, interspace 1.5 cm (2–3 training sessions)	0 - 6 faults or more
	1 - 5 faults
	2 - 4 faults
	3 - 3 faults
	4 - 2 faults
	5 - 1 fault
	6 - no fault step

Before photothrombosis, the animal should achieve 22 points, at day 1 < 11 points, otherwise the animal was excluded from the study.

All animal experiments were approved by the Malmö/Lund ethical committee. Animals were housed in a controlled environment with a 12:12 hour light cycle beginning at 07:00 with room temperature maintained at 22°C and with *ad libitum* access to food and water. For surgery and 2PLSM the animals were anesthetized by inhalation of isoflurane (isoflurane in N_2_O/O_2_ - 0.3:0.7, 4% for induction, 1.8% for maintenance) and local anesthesia (bupivacain 0.25 mg/ml). During anesthesia, the body temperature was monitored by a rectal thermistor probe (Linton Instrumentation, Norfolk, UK) connected to a heating pad maintaining body temperature at 36.7°C.

### Animals

For behavioral analysis, 60 male C57BL/6 mice (28 to 35 g, aged 12 to 16 weeks; Charles River, Sulzfeld, Germany) were purchased. Five out of 60 animals died and nine animals had to be excluded from the study (see below for exclusion criteria). From the 46 remaining animals, 13 mice were treated with AMD3100 after PT (PT/AMD3100), 14 mice were treated with saline after PT (PT/vh), 9 mice were treated with AMD3100 after sham surgery (sham/AMD3100) and 10 mice were treated with saline after sham surgery (sham/vh).

To visualize microglia *in vivo*, 30 male transgenic C57/Bl6 mice that carried GFP under the control of the CX_3_CR1 promotor underwent 2PLSM (aged 30 to 40 weeks, weighing 25 to 35 g, own breeding; breeding pairs were purchased from The Jackson Laboratory; B6.129P(Cg)-Ptprc^a^ Cx3cr1^tm1Litt^/LittJ) [23]. To ensure the presence of CX_3_CR1 and to visualize microglia we only used mice heterozygous for CX_3_CR1 (CX_3_CR1^GFP/+^). It is of note that in addition to brain resident microglia other immune cells express CX_3_CR1 and, therefore, are positive for GFP. Hence, based on previous studies it can be assumed that the majority of cells are microglia investigated in the ischemic territory after PT [24,25]. Four out of these 30 mice had to be excluded due to traumatic injury or lethal anesthesia. The remaining 26 mice were randomized into the following treatment groups: PT/AMD3100, n = 10; PT/vh, n = 8; sham/AMD3100, n = 4; and sham/vh, n = 4. Three AMD3100-treated and one vehicle-treated animal could not be imaged on day 3, but were imaged at the time points before PT, and day 7 and day 14 after PT.

For protein analysis by Western blot, wild-type (n = 10), CX_3_CR1^GFP/+^ (n = 10) and CX_3_CR1^GFP/GFP^ mice (n = 10) were subjected to PT and treated with AMD3100 (0.5 mg/kg twice daily) or vehicle for 5 days starting on day 2 after stroke. On day 6 after PT, FKN levels were determined in the infarct core. Tissue from these animals was also used for analysis of cytokine levels.

### Photothrombosis

PT was carried out as described previously [26,27]. Briefly, anesthetized animals were placed into a stereotactic frame. After shaving, a sagittal skin incision was made on the scalp, subcutaneous connective tissue was removed and the skullbone was dried. Five minutes after intraperitoneal injection of the photosensitizer dye Rose Bengal (0.1 mL at 10 mg/mL; Sigma-Aldrich, Taufkirchen, Germany; Lot#MKBH0535V, the right hemisphere was illuminated with a cold light source (Schott KL 1500 LCD, intensity: 3050 K/4D) through a round aperture measuring 2.5 mm in diameter (equal to an illumination area of 4.909 mm^2^) for 20 minutes (coordinates related to Bregma: +1.5 mm lateral and +0.5 anterior). Thereafter, the scalp incision was sutured and the mice were transferred to their home cage to recover.

For 2PLSM, PT was induced in CX_3_CR1^GFP/+^ transgenic mice. The lesion was located rostral and adjacent to the thinned skull window (for details see below) at the approximate coordinates of 1.5 mm lateral and 0.5 anterior related to bregma. CX_3_CR1^GFP/+^ transgenic animals were illuminated at a light intensity of 3050 K/4E for 15 minutes through a 1.5 mm round aperture (equal to an illumination area of 1.767 mm^2^). Among the CX_3_CR1^GFP/+^ animals undergoing 2PLSM, 2 out of 10 AMD3100 treated (PT/AMD3100) animals and four out of eight vehicle-treated animals (PT/vh) received PT under the conditions 2.5 mm/3050 K/4D/20 minutes. Compared to the parameters in mice with a lesion size of 1.5 mm these PT settings did not affect values for soma size and cell number in the specific region of interest.

### 2-Photon-laser-microscopy

**Figure 2:**
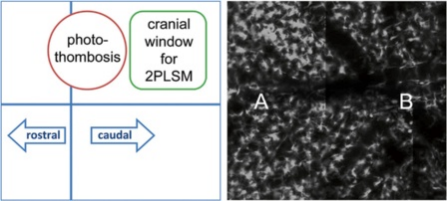
**Illustration of 2-photon-laser-microscopy image (2PLSM) analysis.** In the cartoon to the left the red circle represents the photothrombotic stroke, and the green square the thinned skull-window, through which green fluorescent protein (GFP)-CX_3_CR1^+^ cells were imaged. Close to the infarct we defined region A, representing the peri-infarct zone. The opposite side of the cranial window was defined as region B.

2PLSM was performed in accordance with Yang and collegues [27]. Briefly, after placing the anesthetized animal onto a custom-made plate, a sagittal skin incision from eye to ear level was made and all connective tissue was removed from the skull to attach a custom-made blade to the dry bone (3 M Vetbond-glue, No. 1469SB) so that the head of the animal could be firmly adjusted to a skull-holder on the plate [27]. Using a dental drill and microsurgical blades a thinned window measuring approximately 1.5 × 1.5 mm with a remaining bone thickness of about 20 μm was prepared. The animal was then transferred to the microscope stage and a drop of MilliQ water was placed onto the skull window. After optimization of imaging conditions, 2PLSM imaging was performed in Z-stack planes of 424.9 × 424.9 μm (x/y/z, 0.83 μm/pixel, unless stated otherwise) down to a subdural depth of 75 to 150 μm by a multiphoton Zeiss LSM 7MP upright laser scanning microscope using a W-Plan Apochromat 20_/1.0 DIC Vis-IR water immersion objective (×20, Carl Zeiss, Jena, Germany) controlled by ZEN 2010 imaging software (Carl Zeiss) (Figure 2). GFP was excited by a Mai Tai DeepSee Ti:Sapphire pulsed laser (Spectra-Physics, Irvine, CA, USA) tuned to 875 nm [28]. Further computerized analysis followed *ex vivo* with ImageJ (1.43u, NIH, Washington, USA) and Adobe Photoshop (CS5 extended 12.04x64, Adobe, USA).

We studied number and soma size of GFP^+^ cells (CX_3_CR1^+)^ in the entire area under the thinned skull window and defined a region “A” located adjacent to the infarct core and a region “B” most distant from the infarct core (Figure 2). Microglia were counted and their soma sizes determined at Otsu-threshold [29,30] within regions of interest represented by z-stacks measuring 424.9 × 424.9 × 75 μm (x/y/z) located in either A or B. To eliminate microglia activation caused by any experimental procedures (for example, preparing the skull window or laser stimulation) respective results obtained in sham-operated mice were subtracted from those found in mice subjected to PT. In addition, we defined a value “C” represented by the equation C(dx) = (A(dx) − A(pre)) − (B(dx) − B(pre)) where the increase in number in cells from before infarct induction (pre) to a day x (dx) in region B was subtracted from the respective value in region A. By combined use of sham corrections and this algorithm we gained precise information about the stroke-induced changes in number of microglial cells.

### Infarct size measurement

Brains from perfusion-fixed animals were cut into coronal sections with a thickness of 30 μm and stained for neuronal nuclei (NeuN; procedure described in the immunohistochemistry paragraph). The noninjured portion of the ipsilateral and contralateral hemisphere were encircled and the indirect infarct volume was calculated by integration of areas from serial sections (1 mm) of each brain as described previously [31].

### Immunohistochemistry

Brain sections (30 μm in thickness) were rinsed in phosphate-buffered saline (PBS). Endogenous peroxidase activity was quenched by washing the sections in a solution of PBS with 3% H_2_O_2_ and 10% methanol for 15 minutes. After subsequent rinsing in PBS, blocking was achieved by incubation in a solution containing 5% normal donkey serum (NDS) in 0.25% Triton X-100 in PBS (Tx/PBS) for 1 hour. Thereafter, sections were incubated with a mouse anti-neuronal Nuclei (NeuN) antibody (diluted at 1:1000, Merck Millipore, Billerica, MA, USA) or a rabbit anti-CXCR4 antibody (diluted at 1:200; Abcam, Cambridge, UK) at 4°C in 5% NDS in Tx/PBS overnight. The next day, sections were rinsed in 1% NDS in Tx/PBS followed by incubation with respective biotinylated secondary antibodies in 2% NDS in Tx/PBS at room temperature for 90 minutes. Sections were washed with Tx/PBS and incubated with avidin-biotin-complex (ABC; Vector Laboratories, Burlingame, CA, USA) for 1 hour. Following this incubation, the sections were rinsed in PBS. The ABC reaction was visualized using NiDAB (Dabsafe, Saveen Werner AB, Limhamn, Sweden) with the addition of NiCl_2_ and a mixture of 3% H_2_O_2_ in H_2_O. Thereafter, sections were rinsed in PBS, mounted on glass slides, dried overnight, dehydrated, treated with xylene and then cover-slipped with Pertex (Histolab AB, Västra Frölunda, Sweden).

### Immunofluorescence

Brain sections (thickness 30 μm) from 4% paraformaldehyde-perfused animals were used. After blocking in 5% NDS in Tx/PBS for 60 minutes, sections were incubated with primary antibody in Tx/PBS in 5% NDS at 4°C overnight. The following antibodies were used: rat anti-cluster of differentiation 68 (CD68; diluted at 1:200; AbD Serotec, Düsseldorf, Germany), rabbit anti-CXCR4 (diluted at 1:200; Abcam), mouse anti-CD45 (diluted at 1:25; AbD Serotec) and rabbit anti-ionized calcium binding adaptor molecule 1 (Iba1; diluted 1:2000; Wako Chemicals, Neuss, Germany). The next day, the sections were rinsed with 1% NDS in Tx/PBS three times for 10 minutes. All the following steps were performed in the dark in order to preserve the fluorophores conjugated to the secondary antibodies. Sections were incubated with secondary antibodies in 2% NDS in Tx/PBS for 90 minutes at room temperature and rinsed in PBS three times for 10 minutes. Finally, the sections were rinsed in PBS and mounted on supercharged glass slides, allowed to dry, cover-slipped using PVA-DABCO and analyzed using LSM 510 confocal microscopy (Carl Zeiss).

### Western blotting

Proteins were extracted from brain tissue as described earlier [32]. Proteins were separated on a 10% SDS polyacrylamide gel and were transferred onto polyvinyldifluoride membranes. Unspecific protein binding sites were blocked in blocking buffer containing 20 mM Tris, 136 mM NaCl, pH 7.6, 0.1% Tween 20 and 5% nonfat dry milk. FKN was detected by a rabbit anti-FKN antibody (Torrey Pines Biolabs, Secaucus, NJ, USA; dilution 1:1000). After overnight incubation at 4°C, signals were obtained by binding of a secondary anti-rabbit horse radish peroxidase-linked antibody (1:50000; Sigma-Aldrich, Deisenhofen, Germany) and visualized by exposing the membrane to a charge-coupled device camera (LAS1000, Fujifilm, Tokyo, Japan) using a chemiluminescence kit (Merck Millipore, Billerica, MA, USA). Membranes were stripped and reprobed for β-actin (Sigma-Aldrich), and diluted 1:50,000. After densitometric analysis using Image J software, FKN levels were calculated as a percentage of β-actin expression.

### Measurement of cytokines

Tissue samples from the ischemic core were collected as described previously [32]. Cytokine levels were measured from brain tissue homogenates by a multiplex immunoassay kit according to the manufacturer’s instructions using a SECTOR Imager 6000 reader (Mesoscale, Gaithersburg, MD, USA).

### Statistical analysis

Statistical analysis was performed using Excel® (Microsoft Excel® for Mac 2011, 14.3.6) and SPSS-Statistics® (IBM, Version21, IBM Svenska, Stockholm, Sweden). Results from behavioral testings were evaluated by the Mann–Whitney U-test (significance level 0.05, confidence interval 0.95). Quantity of cells, soma sizes and Western blot results were evaluated by Students *t* test (significance level 0.05, confidence interval 0.95). Cytokine levels were analyzed by one-way analysis of variance. *Post-hoc* multiple comparisons were performed as stated in the figure legends and *P* < 0.05 was considered significant.

## Results

### Treatment with AMD3100 improves functional outcome after photothrombosis in mice without affecting infarct size

**Figure 3:**
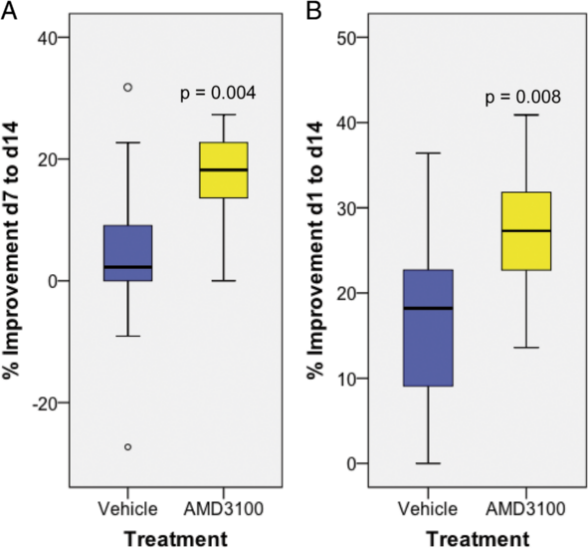
**Impact of AMD3100 treatment on functional recovery after photothrombosis.** Neurological outcome was assessed by a composite neuroscore in CX_3_CR1 wild-type mice before and on days 1, 2, 7 and 14 after photothrombosis (PT). The following numbers of animals were included: PT/AMD3100, n = 13; PT/vehicle, n = 14; sham/AMD3100, n = 9; sham/vehicle, n = 10. **(A)** Percentage of improvement in outcome in mice treated either with saline or AMD3100 from day (d)7 to d14 and **(B)** from d1 to d14 after PT. Statistical analysis was performed by Mann–Whitney U-test, and differences are indicated in the figure.

**Figure 4:**
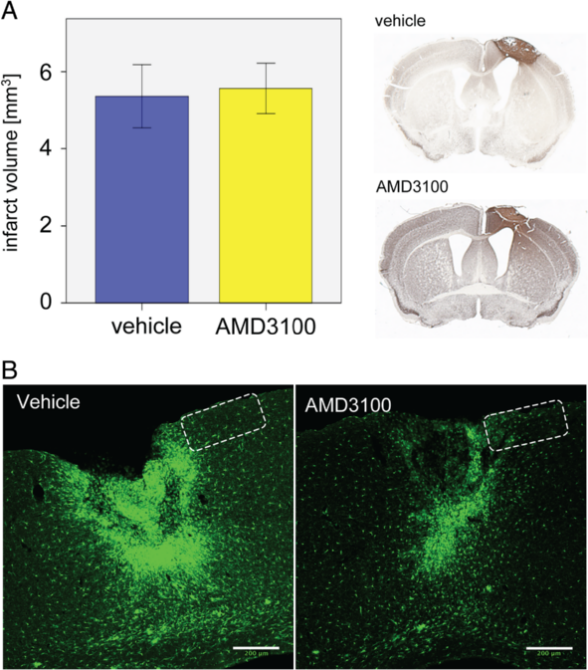
**Effect of AMD3100 treatment on infarct size and accumulation of green fluorescent protein (GFP)-positive cells in the ischemic territory. (A)** Infarct volumes in mice treated with saline (n = 9) or AMD3100 (n = 9) displayed as means ± SEM on day 14 after photothrombosis. Representative coronal sections of a vehicle- and AMD3100-treated animal are shown to the right. **(B)** Accumulation of GFP^+^ cells in the ischemic territory in a vehicle- and an AMD3100-treated CX_3_CR1^+/GFP^ mouse. Scale bars: 200 μm. The frame illustrates the area analyzed for GFP^+^ cells by 2-photon-laser-microscopy.

After permanent focal ischemia induced by PT in wild-type C57BL/6 mice and treatment either with saline or AMD3100, neurological function was assessed by a composite neuroscore before and on day 1, 2, 7 and 14 after PT (Table 1). We observed a significant improvement of the neuroscore in AMD3100-treated mice compared to vehicle-treated animals during the second week after PT (vh 2.3%, AMD3100 18.2%, Mann–Whitney U-test *P* = 0.004; Figure 3A). Over the whole observation period of 14 days, AMD3100-treated animals finally recovered significantly better than vh-treated animals (vh 18.2%, AMD3100 27.3%, Mann–Whitney U-test *P* = 0.008; Figure 3B). Treatment with AMD3100 did not affect behavior in sham-operated mice (data not shown). Importantly, analysis of infarct volumes did not show a significant difference between the treatment groups (vh, 5.35 ± 0.81; AMD3100, 5.56 ± 0.65; Student’s *t* test *P* = 0.85; Figure 4A).

### Treatment with AMD3100 reduces soma size and number of microglia cells in CX_3_CR1^GFP/+^ mice after photothrombosis

**Figure 5:**
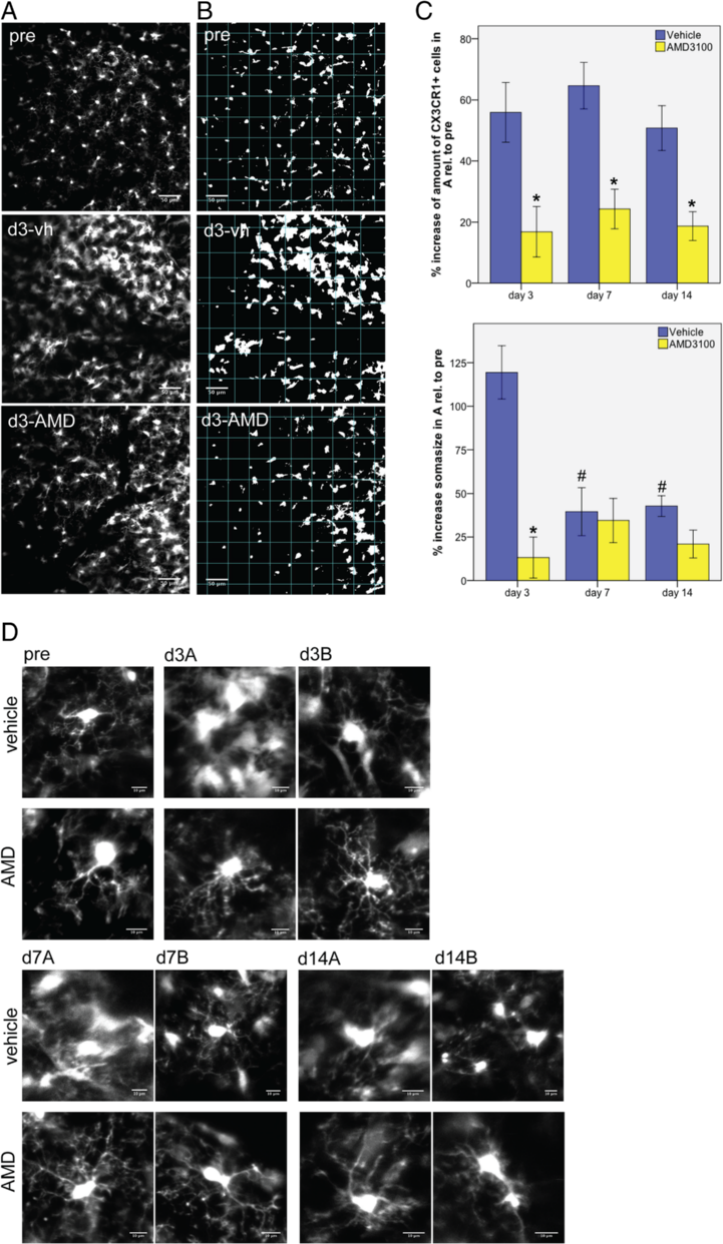
**Analysis of green fluorescent protein (GFP)-positive cells in the peri-infarct area by 2-photon-laser-microscopy (2PLSM). (A)** Representative 2PLSM-image stacks of GFP^+^ microglia measured in a volume of 424.9 × 424.9 × 75 μm (x/y/z) before photothrombosis (PT) (pre) and in the peri-infarct area of a vehicle (d3-vh)- and an AMD3100 (d3-AMD)-treated mouse on day 3 after PT. Scale bars: 50 μm. **(B)** Images displayed in **(A)** after threshold modification according to Otsu [30] in order to measure soma sizes. **(C)** Quantitative analysis of GFP^+^ cells and their soma sizes in the peri-infarct area. Values are normalized to respective values of cell numbers and soma sizes before PT. Error bars represent standard error of the mean. Statistical analysis was performed by Student’s *t* test (*P < 0.05, AMD3100 versus vehicle treatment at the same time point; ^#^ *P* < 0.05, versus the same treatment at day 3). **(D)** Illustration of representative morphologies of GFP^+^ cells in a vehicle- and an AMD3100-treated mouse, respectively, before (pre), and on days 3, 7 and 14 after PT. Scale bars: 10 μm.

PT caused an intensive accumulation of GFP^+^ microglial cells in CX_3_CR1^GFP/+^ mice (Figure 4B). Treatment with AMD3100 strongly attenuated this inflammatory response in the ischemic territory. 2PLSM was carried out to characterize the dynamics during activation of microglial cells in the ischemic territory. We used heterozygous CX_3_CR1^GFP/+^ mice with one allele for GFP and one functional CX_3_CR1 allele. To evaluate the stroke-induced microglial response we studied the number and morphology of GFP^+^ cells accumulating in the peri-infarct area (Figures 2 and 5A-D). By calculating cell numbers within the defined regions A and B we found a significantly lower number of GFP^+^ cells in the proximal peri-infarct area of AMD3100- compared to vehicle-treated animals at days 3, 7 and 14 after PT (Figure 5A-C). Interestingly, this occurred already 1 day after initiating AMD3100 treatment. No changes in the number of cells have been observed within the treatment groups during the observation period of 14 days. Hence, soma sizes were significantly decreased from day 7 onwards in vehicle-treated mice (Figure 5C).

In addition, we observed a clear morphological change of the microglial cells in AMD3100-treated animals, in particular during the early phase after stroke (Figure 5D). At day 3, the microglial somas in vehicle-treated mice more than doubled their size compared to baseline measurements before stroke (increase of soma size: 119.4%) (Figure 5C, D). Soma sizes of microglia in AMD3100-treated mice only increased by 13.2% (Figure 5C, D). At later time points soma sizes were similar, there was no significant difference in the increase of soma size detectable among the treatment groups. Together, our results suggest that treatment with AMD3100 significantly modifies activation of microglia in the peri-infarct area after PT in mice within 24 hours after the first drug administration.

### Green fluorescent protein-positive cells in CX_3_CR1^GFP/+^ mice reveal microglia/macrophage-specific immunohistochemical properties of phagocytosing cells

**Figure 6:**
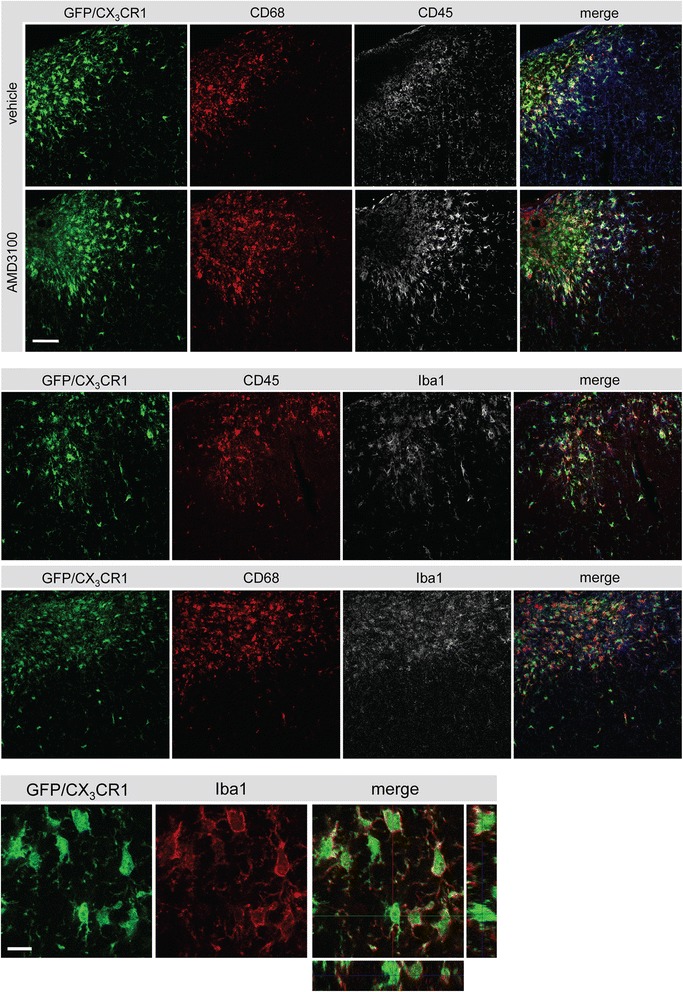
**Phenotype of green fluorescent protein (GFP)-positive cells in the ischemic territory.** Upper panels: colocalization of CD68 (Cy3, red), GFP (green, CX_3_CR1) and CD45 (white, Cy5) in the ischemic territory of a vehicle- and AMD3100-treated mouse after photothrombosis (PT). Middle panels illustrates the expression of ionized calcium binding adaptor molecule 1 (Iba1) in CD68/GFP- and CD45/GFP-positive cells. Scale bar: 50 μm. Lower panel: colocalization of GFP (green) with Iba1 (Cy3, red) in the ischemic core after PT. Scale bar: 10 μm.

To further analyze the phenotype of CX_3_CR1^+^ green fluorescent cells in CX_3_CR1^GFP/+^ mice, we performed immunohistochemistry in the animals that had been sacrificed after 2PLSM-experiments at d14 after stroke. As shown in Figures 4B and 6, accumulation of GFP^+^ cells was observed in the infarct core and the peri-infarct area. The majority of cells also were found positive for Iba1, a marker for activated microglia/macrophages [33] (Figure 6). The distribution of cells in the infarct core was not homogenous, but mostly appeared in a typical amoeboid morphology indicative for phagocytosing cells. For further characterization, co-stainings were performed with CD68, a marker for phagocytosis, and CD45, a marker for peripheral immune cells infiltrating the ischemic territory (Figure 6). GFP^+^ cells were positive for CD68 and CD45 in the infarct core and adjacent peri-infarct area. Some of the cells co-expressed CD68 and CD45. Together, our data show the accumulation of microglia/macrophages in the ischemic territory.

**Figure 7:**
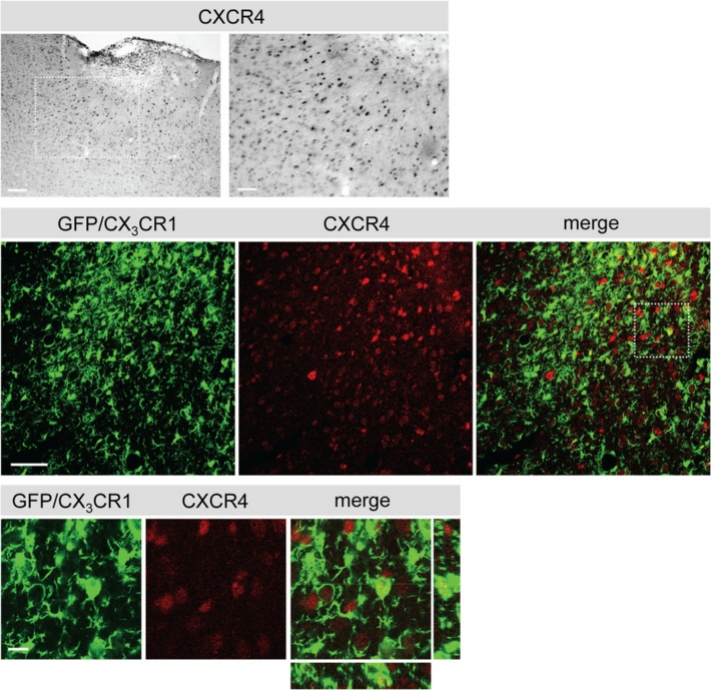
**Phenotype of green fluorescent protein (GFP)-positive cells in the ischemic territory.** Upper panel: expression of CXCR4 in the ischemic territory 14 days after photothrombosis (PT). A higher magnification of the squared area is shown to the right. Scale bars: left 100 μm; right 50 μm. Lower panel: co-staining for CXCR4 (Cy5, red) and GFP (green, CX_3_CR1) in the proximal peri-infarct area. Higher magnification of the squared area is shown in the bottom panel. Scale bars: low magnification 50 μm, high magnification 10 μm.

We further assessed the expression of CXCR4 to find out more about the target cells of AMD3100. In addition to neocortical neurons, CXCR4-positive cells were detected in the ischemic territory (Figure 7). Co-staining of CXCR4 with GFP demonstrates their expression in microglia/macrophages in this region supporting previous studies (Figure 7) [3]. These results illustrate that the majority of GFP^+^ cells accumulating in and around the infarct display microglia/macrophage properties and that these cells might be susceptible for the treatment with AMD3100.

### Reduction of fractalkine levels depends on functional CX_3_CR1 in the subacute post-stroke phase

**Figure 8:**
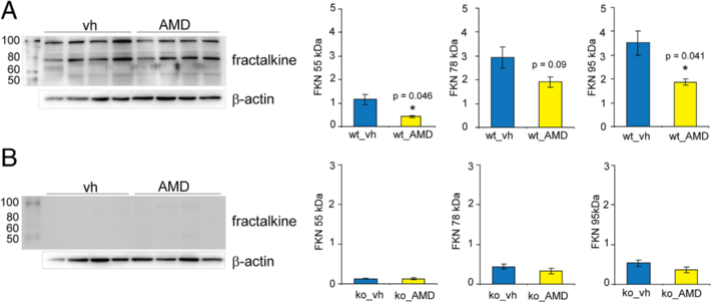
**Effect of AMD3100 treatment on levels of fractalkine (FKN) after photothrombosis. (A)** Levels of FKN in the infarct core of wild-type (wt) and **(B)** CX_3_CR1-deficient (ko) mice treated either with AMD3100 (AMD) or vehicle (vh) for 5 days starting on day 2 after photothrombosis. Specific bands for FKN and β-actin have been quantified, and ratios for the 55 kDa, 78 kDa and 95 kDa FKN are displayed as means ± SEM. Statistical differences are indicated in the graphs and were performed by Student’s *t* test.

FKN is the endogenous ligand for CX_3_CR1 and it has been shown that activation of the CXCL12/CXCR4 pathway induces the release of FKN by increased activity of the protease ADAM10/17 [13]. To test if CXCR4 antagonism affects FKN after PT, wild-type (n = 8), heterozygote (CX_3_CR1^GFP/+^) (n = 8) and CX_3_CR1-deficient (CX_3_CR1^GFP/GFP^) mice (n = 8) were subjected to PT and treated with AMD3100 (0.5 mg/kg twice daily) or vehicle for a total of 5 days. On day 6 after PT, FKN levels were determined in the infarct core by Western blotting. Three specific bands have been identified (Figure 8). While the 95 kDa band corresponds to the released soluble form of FKN, the origin of the 55 kDa and 78 kDa bands have not been unraveled yet. FKN levels found in wild-type animals were decreased in animals treated with AMD3100 compared to mice treated with vehicle. In particular, soluble FKN (95 kDa FKN; vh, 3.51 ± 0.5; AMD3100, 1.87 ± 0.14 arbitrary units (AU)) and the 55 kDa FKN (vh, 1.15 ± 0.22; AMD3100, 0.42 ± 0.04 AU) signal were significantly reduced in AMD3100-treated animals (Figure 8A).

Interestingly, vehicle-treated CX_3_CR1-deficient mice showed lower FKN levels compared to their wild-type littermates, and treatment did not significantly affect the levels of any of the three forms of FKN (Figure 8B). In addition, the levels of FKN did not differ when comparing mice of both genotypes treated with AMD3100 (data not shown). CX_3_CR1^GFP/+^ mice showed a less prominent reduction compared to wild-type animals of the 78 kDa FKN (vh, 2.03 ± 0.16; AMD3100, 1.37 ± 0.13 AU) and the 95 kDa FKN form (vh, 1.92 ± 0.13; AMD3100, 1.45 ± 0.04 AU) while the 55 kDa form (vh, 1.91 ± 0.17; AMD3100, 1.54 ± 0.05 AU) remained unaffected. These results show that treatment with AMD3100 significantly affects the release of FKN in the infarct core, and that the release is dependent on a functional receptor, CX_3_CR1.

### Treatment with AMD3100 reduced the level of pro-inflammatory cytokines in CX_3_CR1 wild-type mice after photothrombosis

**Figure 9:**
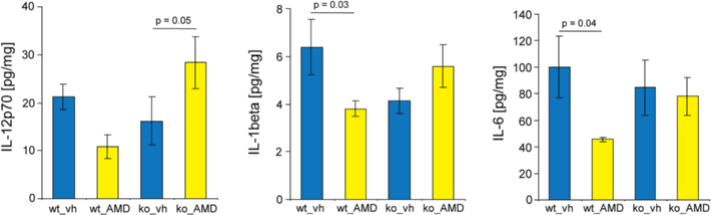
**Cytokine analysis in the infarct core after photothrombosis (PT).** Levels of the cytokines interleukin (IL)-12p70, IL-1β, and IL-6 in the infarct core of wild-type and CX_3_CR1-deficient mice treated either with AMD3100 (0.5 mg/kg) or vehicle (saline) for 5 consecutive days after PT. Five animals are included in each treatment group. Data are presented as means ± standard error of the mean. Statistical differences are indicated in the figure (one-way analysis of variance, Fisher’s least significant difference test).

Levels of pro-inflammatory cytokines were measured in order to study inflammation in the ischemic territory of CX_3_CR1 wild-type and CX_3_CR1-deficient mice subjected to PT. As shown in Figure 9, reduced levels of interleukin (IL)-1β) and IL-6 were found in CX_3_CR1 wild-type mice treated with AMD3100 (0.5 mg/kg) for a total of 5 days (study III) compared to vehicle-treated animals. Interestingly, the levels of IL-12p70, interferon-gamma, IL-2, CXCL1 and IL-5 were also lower in AMD3100-treated mice (data not shown); however, differences did not reach statistical significance. In contrast, treatment with AMD3100 did not affect the levels of cytokines in CX_3_CR1-deficient mice, whereas levels of IL-12p70 were significantly increased compared to vehicle-treated mice (Figure 9). Based on these experiments, we conclude that a functional CX_3_CR1 receptor is essential to achieve anti-inflammatory effects mediated by AMD3100 treatment.

## Discussion

The present investigation demonstrates that treatment with the CXCR4 antagonist AMD3100 in mice subjected to permanent focal ischemia enhances long-term recovery of lost neurological function. Moreover, our data show a correlation between the improved outcome after stroke and a reduced microglial response during the first 14 days. Importantly, treatment did not interfere with the acute tissue response involving cell death mechanisms and the early activation of microglia observed during the first hours after stroke. In addition, we provide the first *in vivo* evidence that levels of FKN, the endogenous ligand for CX_3_CR1, in the ischemic territory are affected by inhibition of the CXCL12/CXCR4 pathway.

### AMD3100 enhances recovery of lost neurological function after photothrombosis

In line with previous investigations showing an improved outcome after AMD3100 treatment in mice subjected to permanent MCAO [34] and rats subjected to transient MCAO [3] we observed better neurological scores in mice subjected to PT treated with AMD3100 for a total of 12 days starting on day 2 after stroke. In contrast to previous studies performed in rats, AMD3100 treatment had no immediate effect on recovery. Differences have not been observed until the second week after stroke onset. This might be due to various mechanisms of recovery dependent on the experimental model (permanent versus transient model) and species, including specific pharmacokinetics in mice and rats. In addition, several studies have demonstrated that application of CXCL12 (stromal cell line derived factor 1, SDF1) exerts beneficial effects on cellular repair mechanism recovery and, especially, the proliferation of neuronal progenitor cells and their migration to the site of injury [35,36]. Hampered CXCL12/CXCR4 signaling by AMD3100 downregulates the migration of neuroblasts at the site of ischemic injury 4 weeks after stroke [37]. In these studies, however, functional outcome after administration of the CXCR4 antagonist AMD3100 has not been assessed. We have shown that administration of AMD3100 during the first days after stroke conveys anti-inflammatory actions [3]. The migration of neuronal progenitor cells from stem cell niches towards the ischemic lesion is relevant for stroke recovery; however, during the early period after stroke and due to multiple ongoing processes, the inhibition of detrimental inflammatory processes prevails and is of prime significance to regain function.

### Microglia activation is downregulated by blockade of CXCL12 actions

Our study supports the idea that downregulation of the FKN/CX_3_CR1 pathway diminishes detrimental actions of post-stroke inflammation. Similar results were observed in the acute phase after transient MCAO showing that CX_3_CR1 deficiency reduced ischemic damage and CX_3_CR1-deficient mice displayed a stroke-protective inflammatory milieu during the first 72 hours after stroke [19,38].

The interaction between microglia, neurons and other surrounding cells after stroke is mainly accomplished by molecular cues such as the release of danger-associated molecule patterns and, more specifically, cytokines/chemokines [39]. Among others, the CXCL12/CXCR4 and the FKN/CX_3_CR1 pathways have been implicated in the activation of immune cells in the post-ischemic brain [12,13,17]. Earlier studies demonstrated an involvement of the FKN/CX_3_CR1 pathway in cell adhesion and chemoattraction of monocytes but also microglia *in vitro* [8,40]. In addition, it has been shown that the application of FKN to microglia cultures reduces their phagocytotic activity [20]. It has therefore been suggested that the activation of CX_3_CR1 by released FKN is involved in the regulation of microglia to remain in a ramified phenotype with potential neuroprotective properties [20,41]. AMD3100 specifically blocks the actions of CXCL12 and thus inhibits the neuronal and glial release of FKN [13,42]. Consequently, an excessive inflammatory response would be expected. According to our observations, however, this is not the case. Also others demonstrated that AMD3100 attenuated the post-stroke inflammatory response during the early phase after transient MCAO in mice and reduced brain edema, which was accompanied by an improved functional outcome [34]. A reduction of inflammation has also been observed in a rat transient MCAO model when treatment was initialized on day 2 after stroke onset [3]. In the present study we observed a lower cell number, a stabilization of morphology of microglia accompanied by reduced levels of pro-inflammatory cytokines indicating that AMD3100 treatment mitigates the post-stroke stimulation of microglial cells. In AMD3100-treated mice, microglia hardly showed any signs of swelling in the early post-stroke phase, and microglia accumulation or proliferation was reduced during the whole observation period of 14 days, possibly due to an initial inhibition of phenotype transformation. Based on previous studies, the majority of GFP-positive cells studied here are microglia cells. However, cells cannot be distinguished from invading macrophages based on the presentation of antigens.

Our results also indicate the necessity of a specific time window to achieve beneficial effects by treatment with AMD3100. To summarize, our data imply that both pathways (CXCL12/CXCR4 and FKN/CX_3_CR1) specifically impact on the activation of microglia and putative intercellular crosstalks. Specifically, we demonstrated that the blockade of CXCL12 actions at the CXCR4 reduced FKN levels, which could be responsible for the observed attenuated microglia activation. It has also been shown that AMD3100 acts as an allosteric agonist on the receptor CXCR7 [43]. Based on our results, we conclude that the effects at CXCR4 are not abrogated by potential binding of AMD3100 to CXCR7. However, we cannot exclude that AMD3100 bound to CXCR7 exerts functions contributing to recovery of function after stroke.

### Phenotype of GFP^+^ cells in the ischemic territory of CX_3_CR1^GFP/+^ mice

Investigation of the phenotype of GFP^+^ immune cells in the ischemic territory showed that 14 days after induction of PT, GFP^+^/CD68^+^ microglia/macrophages accumulated predominantly in the ischemic infarct core. Only a small population of cells was positive for CD45, indicating also the presence of blood-derived leukocytes. Together with an amoeboid morphology these cells most likely represented phagocytes. Co-localization of GFP^+^ cells with Iba1 further supports the idea that these cells were in an activated state as demonstrated previously [33]. Within the infarct core, micrographs also show CD68^+^ cells lacking GFP. A conceivable explanation of this finding could be the existence of different CD68-expressing immune cell populations that may downregulate the expression of GFP driven by the CX_3_CR1 promotor. Further research is needed to explain this phenomenon.

The expression of CXCR4 in the post-ischemic brain confirms a target for AMD3100 treatment after ischemic injury. CXCR4^+^ cells in the infarct core and adjacent peri-infarct area displayed a round-shaped morphology and have been identified as immune cells previously [3]. Other CXCR4^+^ cells observed throughout the brain could be identified as neurons [3,44].

### Relevance of fractalkine for immune cell activation and inflammation after stroke

Several studies have examined the role of FKN after experimental stroke, with conflicting results. Here we demonstrate a significant decrease of FKN levels in the ischemic infarct core of wild-type mice treated with AMD3100 for 5 days. Thus, antagonism of CXCR4 reduces the release of FKN after stroke, which confirms earlier published data [13]. Importantly, immunofluorescence analysis showed CXCR4 expression on immune cells and neuron-like cells in the post-ischemic brain indicating that both cell types are susceptible for AMD3100 treatment and may release FKN.

In mice deficient for CX_3_CR1, FKN levels were lower than those obtained in wild-type littermates. A possible explanation might be a positive feedback loop between glia and neurons regulating FKN shedding. Moreover, treatment with AMD3100 did not affect the already reduced levels of FKN in CX_3_CR1-deficient mice subjected to PT. We therefore propose that a functional receptor, CX_3_CR1, is necessary for FKN modulation through CXCR4 antagonism.

It also has been shown that in CX_3_CR1-deficient mice there might be a lower production of pro-inflammatory cytokines, leading to a protective inflammatory milieu in stroke with smaller lesions [19,45]. However, one has to consider that smaller infarct sizes *per se* may result in lower levels of pro-inflammatory molecules. In the present study we could not find any differences concerning the levels of pro-inflammatory cytokines in the infarct core after PT in wild-type compared to CX_3_CR1-deficient mice. On the other hand, our results indicate that anti-inflammatory effects caused by AMD3100 treatment require a functional CX_3_CR1 since the drug was ineffective in deficient mice.

We demonstrate that reduced endogenous FKN levels and reduced levels of pro-inflammatory cytokines are accompanied by an enhanced recovery of lost neurological function. This is in contrast to studies showing that FKN significantly reduced brain damage after permanent focal ischemia [20]. Discrepancies might be explained by differences in the study designs. While the present project investigated the role of endogenous FKN, recombinant FKN was administered to mice shortly before induction of stroke in the previous study and infarct volumes have been studied at an earlier time point after stroke [20]. Nevertheless, it could be speculated that an increase of FKN in the early phase after stroke has different effects on inflammatory cascades and cell death mechanisms than at later stages. Acutely after stroke there are more brain resident CX_3_CR1^+^ microglial cells present in the ischemic territory that are responsive to FKN. Several days later, the tissue milieu in the ischemic territory changes, with accumulation of different subsets of CX_3_CR1-expressing immune cells [23,46].

## Conclusion

Our study shows that treatment with the CXCR4 antagonist AMD3100 significantly enhanced long-term recovery of lost neurological function after permanent focal ischemia. Treatment with AMD3100 inhibited the activation of microglia in the proximal peri-infarct area most likely due to reduced levels of FKN during the first week after stroke. Results support the idea that specific CXCR4 antagonism might be exploited in future approaches to improve rehabilitative treatment in stroke patients.

## Abbreviations

2PLSM: 2-photon-laser-microscopy
ABC: Avidin-biotin-complex
AMD3100: 1′-[1,4-phenylenebis(methylene)]bis[1,4,8,11-tetraazacyclotetradecane]
AU: Arbitrary units
CXCL: C-X-C chemokine ligand
CXCR: C-X-C chemokine receptor
FKN: Fractalkine
GFP: Green fluorescent protein
IL: Interleukin
MCAO: Middle cerebral artery occlusion
NDS: Normal donkey serum
PBS: Phosphate-buffered saline
PT: Photothrombosis
Tx: Triton X-100
vh: Vehicle

## References

[1] Dancause N, Nudo RJ (2011). Shaping plasticity to enhance recovery after injury. Prog Brain Res..

[2] Brewer L, Horgan F, Hickey A, Williams D (2013). Stroke rehabilitation: recent advances and future therapies. QJM..

[3] Ruscher K, Kuric E, Liu Y, Walter HL, Issazadeh-Navikas S, Englund E (2013). Inhibition of CXCL12 signaling attenuates the postischemic immune response and improves functional recovery after stroke. J Cereb Blood Flow Metab..

[4] Subramanian S, Zhang B, Kosaka Y, Burrows GG, Grafe MR, Vandenbark AA (2009). Recombinant T cell receptor ligand treats experimental stroke. Stroke..

[5] Saleh A, Schroeter M, Jonkmanns C, Hartung HP, Modder U, Jander S (2004). In vivo MRI of brain inflammation in human ischaemic stroke. Brain..

[6] Cramer SC (2008). Repairing the human brain after stroke. II Restorative therapies. Ann Neurol..

[7] Denes A, Ferenczi S, Kovacs KJ (2011). Systemic inflammatory challenges compromise survival after experimental stroke via augmenting brain inflammation, blood–brain barrier damage and brain oedema independently of infarct size. J Neuroinflammation..

[8] Chapman GA, Moores K, Harrison D, Campbell CA, Stewart BR, Strijbos PJ (2000). Fractalkine cleavage from neuronal membranes represents an acute event in the inflammatory response to excitotoxic brain damage. J Neurosci..

[9] Harrison JK, Jiang Y, Chen S, Xia Y, Maciejewski D, McNamara RK (1998). Role for neuronally derived fractalkine in mediating interactions between neurons and CX3CR1-expressing microglia. Proc Natl Acad Sci U S A..

[10] Hatori K, Nagai A, Heisel R, Ryu JK, Kim SU (2002). Fractalkine and fractalkine receptors in human neurons and glial cells. J Neurosci Res..

[11] Bazan JF, Bacon KB, Hardiman G, Wang W, Soo K, Rossi D (1997). A new class of membrane-bound chemokine with a CX3C motif. Nature..

[12] Tarozzo G, Campanella M, Ghiani M, Bulfone A, Beltramo M (2002). Expression of fractalkine and its receptor, CX3CR1, in response to ischaemia-reperfusion brain injury in the rat. Eur J Neurosci..

[13] Cook A, Hippensteel R, Shimizu S, Nicolai J, Fatatis A, Meucci O (2010). Interactions between chemokines: regulation of fractalkine/CX3CL1 homeostasis by SDF/CXCL12 in cortical neurons. J Biol Chem..

[14] Cardona AE, Pioro EP, Sasse ME, Kostenko V, Cardona SM, Dijkstra IM (2006). Control of microglial neurotoxicity by the fractalkine receptor. Nat Neurosci..

[15] Jung S, Aliberti J, Graemmel P, Sunshine MJ, Kreutzberg GW, Sher A (2000). Analysis of fractalkine receptor CX(3)CR1 function by targeted deletion and green fluorescent protein reporter gene insertion. Mol Cell Biol..

[16] Nishiyori A, Minami M, Ohtani Y, Takami S, Yamamoto J, Kawaguchi N (1998). Localization of fractalkine and CX3CR1 mRNAs in rat brain: does fractalkine play a role in signaling from neuron to microglia?. FEBS Lett..

[17] Meucci O, Fatatis A, Simen AA, Miller RJ (2000). Expression of CX3CR1 chemokine receptors on neurons and their role in neuronal survival. Proc Natl Acad Sci U S A..

[18] Boehme SA, Lio FM, Maciejewski-Lenoir D, Bacon KB, Conlon PJ (2000). The chemokine fractalkine inhibits Fas-mediated cell death of brain microglia. J Immunol..

[19] Denes A, Ferenczi S, Halasz J, Kornyei Z, Kovacs KJ (2008). Role of CX3CR1 (fractalkine receptor) in brain damage and inflammation induced by focal cerebral ischemia in mouse. J Cereb Blood Flow Metab..

[20] Cipriani R, Villa P, Chece G, Lauro C, Paladini A, Micotti E (2011). CX3CL1 is neuroprotective in permanent focal cerebral ischemia in rodents. J Neurosci..

[21] Zhang L, Schallert T, Zhang ZG, Jiang Q, Arniego P, Li Q (2002). A test for detecting long-term sensorimotor dysfunction in the mouse after focal cerebral ischemia. J Neurosci Methods..

[22] Farr TD, Liu L, Colwell KL, Whishaw IQ, Metz GA (2006). Bilateral alteration in stepping pattern after unilateral motor cortex injury: a new test strategy for analysis of skilled limb movements in neurological mouse models. J Neurosci Methods..

[23] Kucharz K, Wieloch T, Toresson H (2011). Rapid fragmentation of the endoplasmic reticulum in cortical neurons of the mouse brain in situ following cardiac arrest. J Cereb Blood Flow Metab..

[24] Gelderblom M, Leypoldt F, Steinbach K, Behrens D, Choe CU, Siler DA (2009). Temporal and spatial dynamics of cerebral immune cell accumulation in stroke. Stroke..

[25] Kuric E, Ruscher K (2014). Dynamics of major histocompatibility complex class II-positive cells in the postischemic brain - influence of levodopa treatment. J Neuroinflammation..

[26] Watson BD, Dietrich WD, Busto R, Wachtel MS, Ginsberg MD (1985). Induction of reproducible brain infarction by photochemically initiated thrombosis. Ann Neurol..

[27] Schroeter M, Jander S, Stoll G (2002). Non-invasive induction of focal cerebral ischemia in mice by photothrombosis of cortical microvessels: characterization of inflammatory responses. J Neurosci Methods..

[28] Yang G, Pan F, Parkhurst CN, Grutzendler J, Gan WB (2010). Thinned-skull cranial window technique for long-term imaging of the cortex in live mice. Nature Protoc..

[29] Kozlowski C, Weimer RM (2012). An automated method to quantify microglia morphology and application to monitor activation state longitudinally in vivo. PLoS One..

[30] Otsu N (1979). A threshold selection method from gray-level histograms. IEEE Trans Syst Man Cybern.

[31] Swanson RA, Morton MT, Tsao-Wu G, Savalos RA, Davidson C, Sharp FR (1990). A semiautomated method for measuring brain infarct volume. J Cereb Blood Flow Metab..

[32] Ruscher K, Shamloo M, Rickhag M, Ladunga I, Soriano L, Gisselsson L (2011). The sigma-1 receptor enhances brain plasticity and functional recovery after experimental stroke. Brain..

[33] Ito D, Tanaka K, Suzuki S, Dembo T, Fukuuchi Y (2001). Enhanced expression of Iba1, ionized calcium-binding adapter molecule 1, after transient focal cerebral ischemia in rat brain. Stroke..

[34] Huang J, Li Y, Tang Y, Tang G, Yang GY, Wang Y (2012). CXCR4 antagonist AMD3100 protects blood–brain barrier integrity and reduces inflammatory response after focal ischemia in mice. Stroke..

[35] Ohab JJ, Fleming S, Blesch A, Carmichael ST (2006). A neurovascular niche for neurogenesis after stroke. J Neurosci..

[36] Zendedel A, Nobakht M, Bakhtiyari M, Beyer C, Kipp M, Baazm M (2012). Stromal cell-derived factor-1 alpha (SDF-1alpha) improves neural recovery after spinal cord contusion in rats. Brain Res..

[37] Thored P, Arvidsson A, Cacci E, Ahlenius H, Kallur T, Darsalia V (2006). Persistent production of neurons from adult brain stem cells during recovery after stroke. Stem Cells..

[38] Fumagalli S, Perego C, Ortolano F, De Simoni MG (2013). CX3CR1 deficiency induces an early protective inflammatory environment in ischemic mice. Glia..

[39] Hanisch UK (2013). Proteins in microglial activation - inputs and outputs by subsets. Curr Prot Pept Sci..

[40] Chapman GA, Moores KE, Gohil J, Berkhout TA, Patel L, Green P (2000). The role of fractalkine in the recruitment of monocytes to the endothelium. Eur J Pharmacol..

[41] Kierdorf K, Prinz M (2013). Factors regulating microglia activation. Front Cell Neurosci..

[42] Mizuno T, Kawanokuchi J, Numata K, Suzumura A (2003). Production and neuroprotective functions of fractalkine in the central nervous system. Brain Res..

[43] Kalatskaya I, Berchiche YA, Gravel S, Limberg BJ, Rosenbaum JS, Heveker N (2009). AMD3100 is a CXCR7 ligand with allosteric agonist properties. Mol Pharmacol..

[44] Chalasani SH, Baribaud F, Coughlan CM, Sunshine MJ, Lee VM, Doms RW (2003). The chemokine stromal cell-derived factor-1 promotes the survival of embryonic retinal ganglion cells. J Neurosci..

[45] Fumagalli S, Coles JA, Ejlerskov P, Ortolano F, Bushell TJ, Brewer JM (2011). In vivo real-time multiphoton imaging of T lymphocytes in the mouse brain after experimental stroke. Stroke..

[46] Liesz A, Zhou W, Mracsko E, Karcher S, Bauer H, Schwarting S (2011). Inhibition of lymphocyte trafficking shields the brain against deleterious neuroinflammation after stroke. Brain..

